# PD‐1/PD‐L1 based immunochemotherapy versus chemotherapy alone for advanced esophageal squamous cell carcinoma: A meta‐analysis focus on PD‐L1 expression level

**DOI:** 10.1002/cnr2.1794

**Published:** 2023-05-18

**Authors:** Zixian Jin, Jiping Wang, Jiajing Sun, Chengchu Zhu, Jian Zhang, Bo Zhang

**Affiliations:** ^1^ Key Laboratory of Minimally Invasive Techniques & Rapid Rehabilitation of Digestive System Tumor of Zhejiang Province Taizhou Hospital of Zhejiang Province affiliated to Wenzhou Medical University Linhai China; ^2^ Department of Cardiothoracic Surgery Taizhou Hospital of Zhejiang Province affiliated to Wenzhou Medical University Linhai China; ^3^ Department of Cardiothoracic Surgery Taizhou Hospital of Zhejiang Province, Zhejiang University Linhai China

**Keywords:** PD‐1, PD‐L1, immunochemotherapy, survival, ESCC

## Abstract

**Background:**

Immunochemotherapy has become a new treatment for advanced esophageal squamous cell carcinoma (ESCC).

**Aims:**

We aimed to study the clinical efficacy and toxicity of immunochemotherapy based on PD‐1/PD‐L1 compared with chemotherapy alone in the treatment of advanced ESCC, focusing on analyzing the influence of PD‐L1 expression level.

**Methods and Results:**

Five randomized controlled trials comparing PD‐1/PD‐L1 based immunochemotherapy with chemotherapy alone for advanced ESCC were included. We extracted efficacy data (objective response rate [ORR], disease control rate [DCR], overall survival [OS] rate, progression‐free survival [PFS] rate) and safety data (treatment‐related adverse events, treatment‐related mortality) and performed meta‐analyses. Compared with chemotherapy alone, the ORR and DCR of immunochemotherapy increased by 2.05 times and 1.54 times, respectively. Overall, patients receiving immunochemotherapy had a significant long‐term survival advantage (OS: hazard ratio [HR] = 0.68, 95% confidence intervals [CI] 0.61–0.75; PFS: HR = 0.62, 95% CI 0.55, 0.70, respectively). Even with PD‐L1 tumor proportion score <1%, immunochemotherapy also showed a significant survival advantage (OS: HR = 0.65, 95% CI 0.46–0.93; PFS: HR = 0.56, 95% CI 0.46–0.69, respectively). However, for PD‐L1 combined positive score (CPS) < 1, the survival advantage of immunochemotherapy was not significant (OS: HR = 0.89, 95% CI 0.42–1.90; PFS: HR = 0.71, 95% CI 0.47–1.08, respectively). The toxicity of immunochemotherapy was higher than that of chemotherapy alone, but there was no statistical difference in treatment‐related mortality (odds ratio = 1.11, 95% CI 0.67–1.83).

**Conclusion:**

In this study, treatment‐related mortality was similar between immunochemotherapy and chemotherapy. PD‐1/PD‐L1 based immunochemotherapy significantly could improve survival outcomes in patients with advanced ESCC. For patients with CPS <1, the survival advantage of immunochemotherapy was not significant compared with chemotherapy.

**Abbreviations**
CI: confidence intervals
CPS: combined positive score
CTLA‐4: cytotoxic T‐lymphocyte‐associated antigen‐4
DCR: disease control rate
EAC: esophageal adenocarcinoma
ESCC: esophageal squamous cell carcinoma
HR: hazard ratios
irAE: immune‐related adverse event
OR: odds ratio
ORR: objective response rate
OS: overall survival
PD‐1: programmed cell death 1
PD‐L1: programmed cell death 1 ligand 1
PFS: progression‐free survival
RCT: randomized controlled trial
TPS: tumor proportion score
TRAE: treatment‐related adverse event
TRM: treatment‐related mortality

## 1 INTRODUCTION

Squamous cell carcinoma is one of the main subtypes of esophageal cancer, and the prognosis is still unsatisfactory.^1^, ^2^ The early stage of esophageal cancer is often overlooked due to the lack of distinctive clinical features. By the time patients present with typical symptoms (e.g., progressive dysphagia, retrosternal pain), the disease is often locally advanced or even terminal. When the disease progresses to an advanced stage, surgery is no longer able to cure it, and the survival of the patient is greatly threatened.

In the past, the treatment of advanced esophageal squamous cell carcinoma (ESCC) depended on the entire body of chemotherapy, radiation therapy, and targeted therapy, but the effect was not ideal.^3^, ^4^, ^5^ Immunotherapy is a new treatment option that has shown encouraging efficacy in many cancers.^6^, ^7^ With the advent of immunotherapy era, the treatment of advanced esophageal cancer is gradually changing. Clinically, there are two main immunotherapy options for patients with esophageal cancer, namely, anti‐programmed cell death 1 (anti‐PD‐1)/anti‐programmed cell death 1 ligand 1 (anti‐PD‐L1) and anti‐cytotoxic T‐lymphocyte‐associated antigen‐4 (anti‐CTLA‐4) therapy.^8^ Phase I/II studies in pilot trials have demonstrated antitumor activity and safety of PD‐1/L1 based immunotherapy in patients with unresectable advanced or recurrent esophageal cancer or gastroesophageal junction cancer.^9^, ^10^, ^11^, ^12^, ^13^, ^14^, ^15^ Malignant tumors, including esophageal cancer, can express PD‐L1 binding to PD‐1, which initiates programmed death of T lymphocytes, thereby reducing the effector activity of T lymphocytes and terminating the immune response.^16^ PD‐1/PD‐L1 based immune checkpoint inhibitors can block the PD‐1/PD‐L1 pathway, reactivate tumor‐specific cytotoxic T lymphocytes in the tumor microenvironment, and restore anti‐tumor immune activity.^17^ With the deepening of the cognition of immunotherapy, immunochemotherapy, radiotherapy and even double immunotherapy are gradually derived, and their effects are exciting.^18^, ^19^, ^20^ However, the clinical application of immune checkpoint inhibitors is still in its early stages. Compared with other treatments, the survival benefit of immunotherapy has also been widely concerned.

Since the release of KEYNOTE‐590 trial results, immunochemotherapy has officially entered the first‐line treatment of advanced esophageal cancer.^21^ Currently, the results of a number of clinical trials comparing immunochemotherapy versus chemotherapy alone have been published.^22^, ^23^, ^24^, ^25^ For ESCC patients as a whole, immunochemotherapy had better long‐term outcomes than chemotherapy in all clinical trials. Compared with chemotherapy, Camrelizumab combined with chemotherapy reduced patients' risk of death by 30% and disease progression by 44% (ESCORT‐1st);^22^ Nivolumab combined with chemotherapy reduced the risk of death by 26% and disease progression by 19% (CheckMate‐648);^23^ Sintilimab combined with chemotherapy reduced the risk of death by 37% and disease progression by 44% (ORIENT‐15);^24^ Toripalimab combined with chemotherapy both reduced patients' risk of death and disease progression by 42% (JUPITER‐06).^25^ These studies also showed that patients with advanced ESCC who received immunochemotherapy had significantly higher ORR than those who received chemotherapy alone.

However, as a biomarker, PD‐1/PD‐L1 was not expressed at exactly the same level in all patients. Studies showed that there were differences in the efficacy of immunochemotherapy in subgroups with different PD‐L1 expression levels.^23^, ^24^ This difference means that PD‐1/PD‐L1 based immunochemotherapy may not be appropriate for all ESCC patients. In this meta‐analysis, we attempted to comprehensively analyze the efficacy and safety of PD‐1/PD‐L1 based immunochemotherapy for advanced ESCC, and to evaluate the effect of PD‐L1 expression level on the treatment outcome.

## 2 MATERIALS AND METHODS

### 2.1 Literature retrieval

The search and screening procedures followed the Preferred Reporting Items for Systematic reviews and Meta‐Analyses for Protocols (PRISMA) (Figure S1).^26^ We performed keyword searches in electronic databases of PubMed, Cochrane Library, Web of Science, and EMBASE to identify all relevant records. In addition, conference abstracts published by the International Society for Diseases of the Esophagus (ISDE), the European Society of Medical Oncology (ESMO), and the American Society of Clinical Oncology (ASCO) are also included in our search. Search terms included “esophageal, esophageal carcinoma, esophageal cancer, esophageal squamous cell carcinoma, esophageal malignancy,” “PD‐1, PD‐L1, immunotherapy, immunochemotherapy,” and “pembrolizumab, nivolumab, avelumab, atezolizumab, durvalumab, camrelizumab, toripalimab, sintilimab,” language limited to English and the deadline was August 31, 2022. In addition, we searched references of relevant published studies and review articles to supplement the insufficient keyword retrieval.

### 2.2 Inclusion and exclusion criteria

Study inclusion criteria: (1) Randomized controlled trial (RCT); (2) Patients received chemotherapy combined with immunotherapy based on PD‐1/PD‐L1 or chemotherapy alone; (3) Advanced ESCC was diagnosed; (4) The outcome of interest is efficacy and toxicity; and (5) Studies published in English. Study exclusion criteria: (1) Retrospective studies and non‐randomized controlled clinical trials; (2) Patients with a history of any other malignancy were included in the study. If the results of a clinical trial were published in different journals or in different years, the article with the most complete data were selected.

### 2.3 Data extraction

Two independent authors recorded basic information about each study, including study name, intervention, year of publication, sample size, trial phase, and pathology. Objective response rate (ORR), disease control rate (DCR), progression‐free survival (PFS), overall survival (OS), hazard ratios (HR), 95% confidence intervals (CI) and safety data (treatment‐related adverse event [TRAE], immune‐related adverse event [irAE], treatment‐related mortality [TRM]) were extracted. A more in‐depth subgroup analysis of PFS and OS was also performed according to the expression of PD‐L1, namely tumor proportion score (TPS), and combined positive score (CPS).

### 2.4 Assessment of risk of bias

The overall risk of bias of the literature was assessed according to the Cochrane Handbook of systematic reviews.^27^ The Cochrane Handbook of systematic reviews consists of seven parts: random sequence generation, allocation hiding, the blindness of participants and personnel, blindness of result evaluations, incomplete result data, selective result reporting, and other sources of bias. Two independent authors evaluated the included articles, and the other author made the final decision on controversial sections.

### 2.5 Statistical methods

For time‐survival variables (OS and PFS), HR and 95% CI were extracted to calculate logHR and SE. For categorical variables, such as ORR, DCR and safety events (TRAEs, irAEs and TRM), the number of events and sample size were extracted. Meta‐analyses were performed using random‐effects model in Revman software. The I^2^ test was used to calculate inter‐study heterogeneity and *p*‐value of heterogeneity. If inter‐study heterogeneity was too high (I^2^ > 50%, *p* < .05), sensitivity analysis was further performed to assess the robustness of the meta‐analysis and determine the source of heterogeneity.

## 3 RESULTS

### 3.1 Literature results

Figure S1 shows the literature retrieval and screening process. Five RCTs were included, all of which were multi‐center phase III clinical trials with a total of 2962 patients (Table 1).^21^, ^22^, ^23^, ^24^, ^25^ The KEYNOTE‐590 trial included ESCC and esophageal adenocarcinoma (EAC), from which we could extract relevant data on ESCC. The overall risk of bias was assessed according to the Cochrane Handbook for systematic reviews of interventions, and all the studies were of high quality (Table S1).

**TABLE 1:** Specific data of five randomized controlled trials.

Study	Year	Histology	ICI	N1	N2	OS		PFS		TRAE	irAE	TRD
						HR	95% CI	HR	95% CI			
KEYNOTE‐590	2021	ESCC	PEMBRO	274	274	0.72	0.60–0.88	0.65	0.54–0.78	364 versus 360	95 versus 43	9 versus 5
KEYNOTE‐590	2021	EAC	PEMBRO	99	102	0.74	0.54–1.02	0.63	0.46–0.87			
ESCORT‐1st	2021	ESCC	CAMRE	298	298	0.70	0.56–0.88	0.56	0.46–0.68	296 versus 288	252 versus 98	9 versus 11
CheckMate‐648	2022	ESCC	NIVO	321	324	0.74	0.58–0.96	0.81	0.64–1.04	297 versus 275	‐	5 versus 6
ORIENT‐15	2022	ESCC	SINTI	327	332	0.63	0.51–0.78	0.56	0.46–0.68	321 versus 326	155 versus 81	9 versus 6
JUPITER‐06	2022	ESCC	TORIPA	257	257	0.58	0.43–0.78	0.58	0.46–0.74	183 versus 158	95 versus 68	1 versus 2

Abbreviations: 95% CI, 95% confidence interval; CAMRE, camrelizumab; EAC, esophageal adenocarcinoma; ESCC, esophageal squamous cell carcinomas; HR, hazard ratio; ICI, immune checkpoint inhibitor; irAE, immune‐related adverse event; N1, the number of immunochemotherapy patients; N2: the number of chemotherapy patients; NIVO, nivolumab; OS, overall survival; PEMBRO, pembrolizumab; PFS: progression‐free survival; SINTI, sintilimab; TORIPA, toripalimab; TRAE, treatment‐related adverse event; TRD, treatment‐related death.

### 3.2 ORR and DCR

**FIGURE 1:**
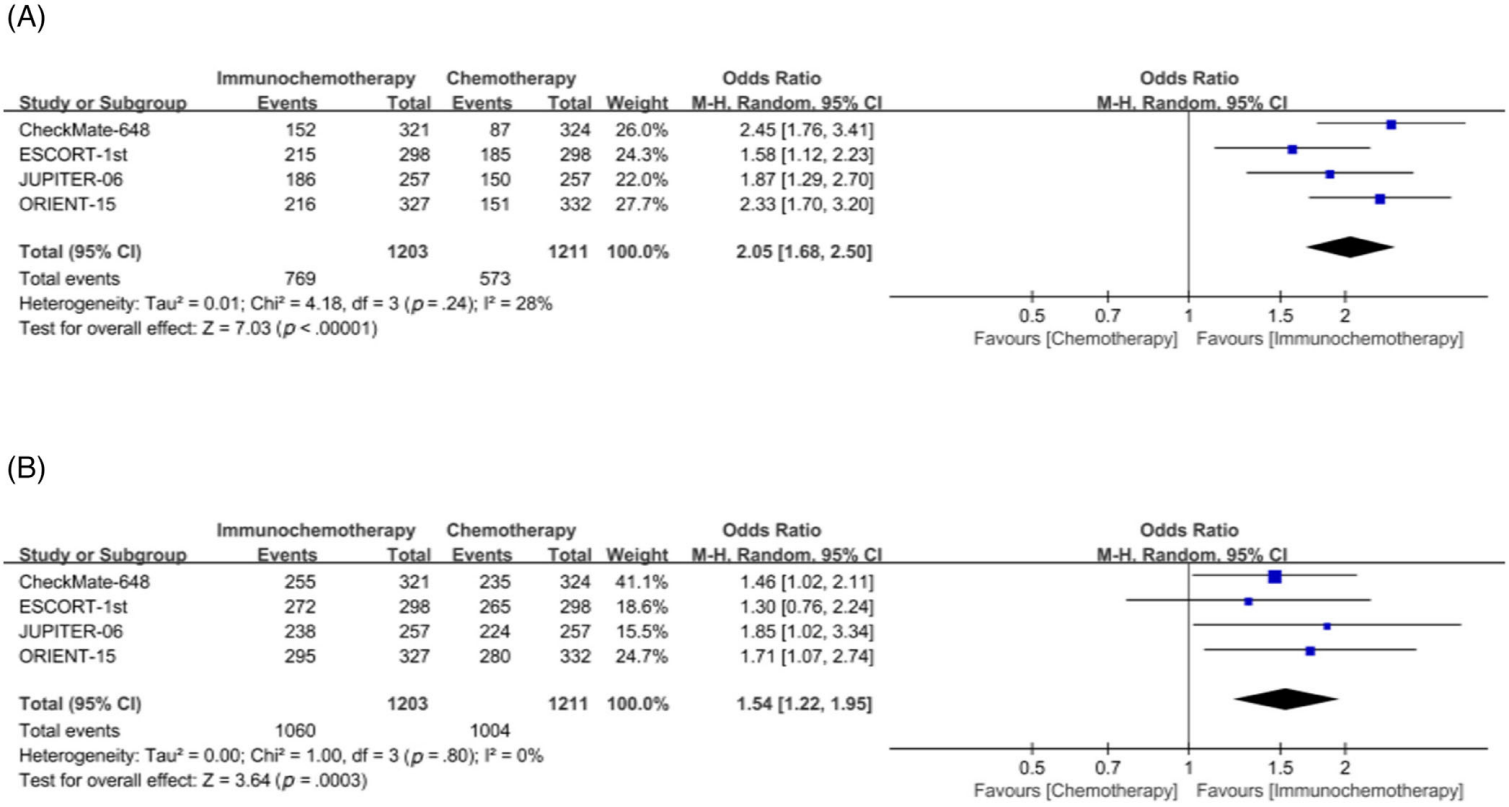
Forest plot of objective response rate (ORR) (A) and disease control rate (DCR) (B) comparison between immunochemotherapy and chemotherapy.

Overall, the ORR was significantly higher in patients who received immunochemotherapy than in patients who received chemotherapy alone. Meta‐analysis showed that the ORR was ~2.05 times higher in the combination group than in the chemotherapy alone group (odds ratio (OR) = 2.05, 95% CI 1.68–2.50) (Figure 1A). Heterogeneity test I^2^ = 28%, *p* = .24, indicating no significant heterogeneity between studies.

Disease control rates were significantly higher in the immunochemotherapy group than in the chemotherapy group alone, ~1.54 times higher (OR = 1.54, 95% CI 1.22–1.95) (Figure 1B). Similarly, according to the results of heterogeneity test, there was no significant heterogeneity between studies (I^2^ = 0, *p* = .80).

### 3.3 OS and PFS

**FIGURE 2:**
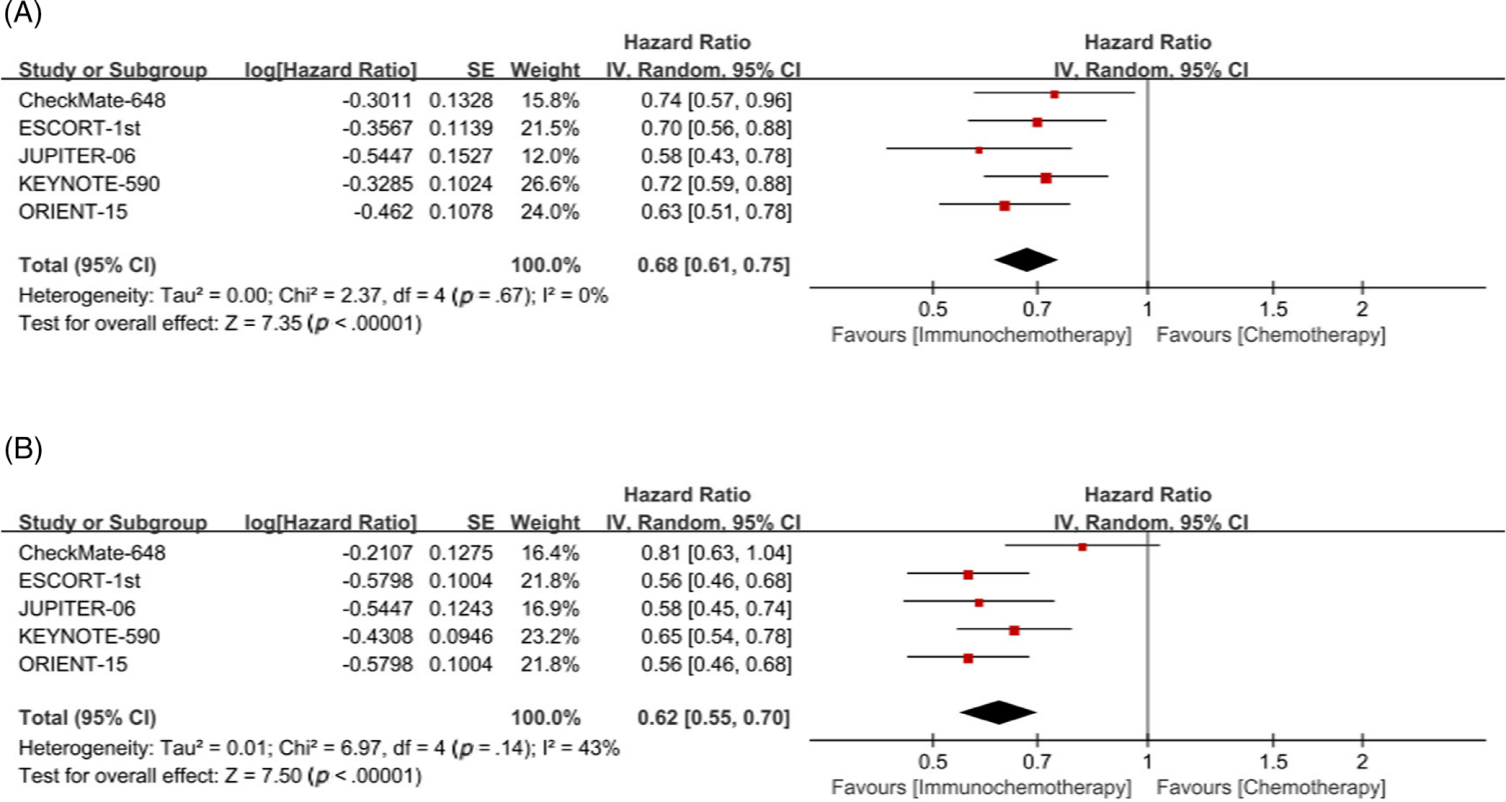
Forest plot of overall survival (OS) (A) and progression‐free survival (PFS) (B) comparison between immunochemotherapy and chemotherapy, when PD‐L1 expression levels were not differentiated.

All of the included clinical trials showed higher long‐term survival rates with immunochemotherapy in advanced ESCC patients compared with chemotherapy alone, when PD‐L1 expression levels were not differentiated. The meta‐analysis showed that immunochemotherapy reduced the overall risk of death by 32% and the risk of disease progression or death by 38% in advanced ESCC patients compared with chemotherapy (OS: HR = 0.68, 95% CI 0.61–0.75; PFS: HR = 0.62, 95% CI 0.55–0.70, respectively) (Figure 2A,B). There was no significant heterogeneity was found between studies comparing OS and PFS (I^2^ = 0, *p* = .67; I^2^ = 43%, *p* = .14, respectively).

### 3.4 Stratified according to TPS

**FIGURE 3:**
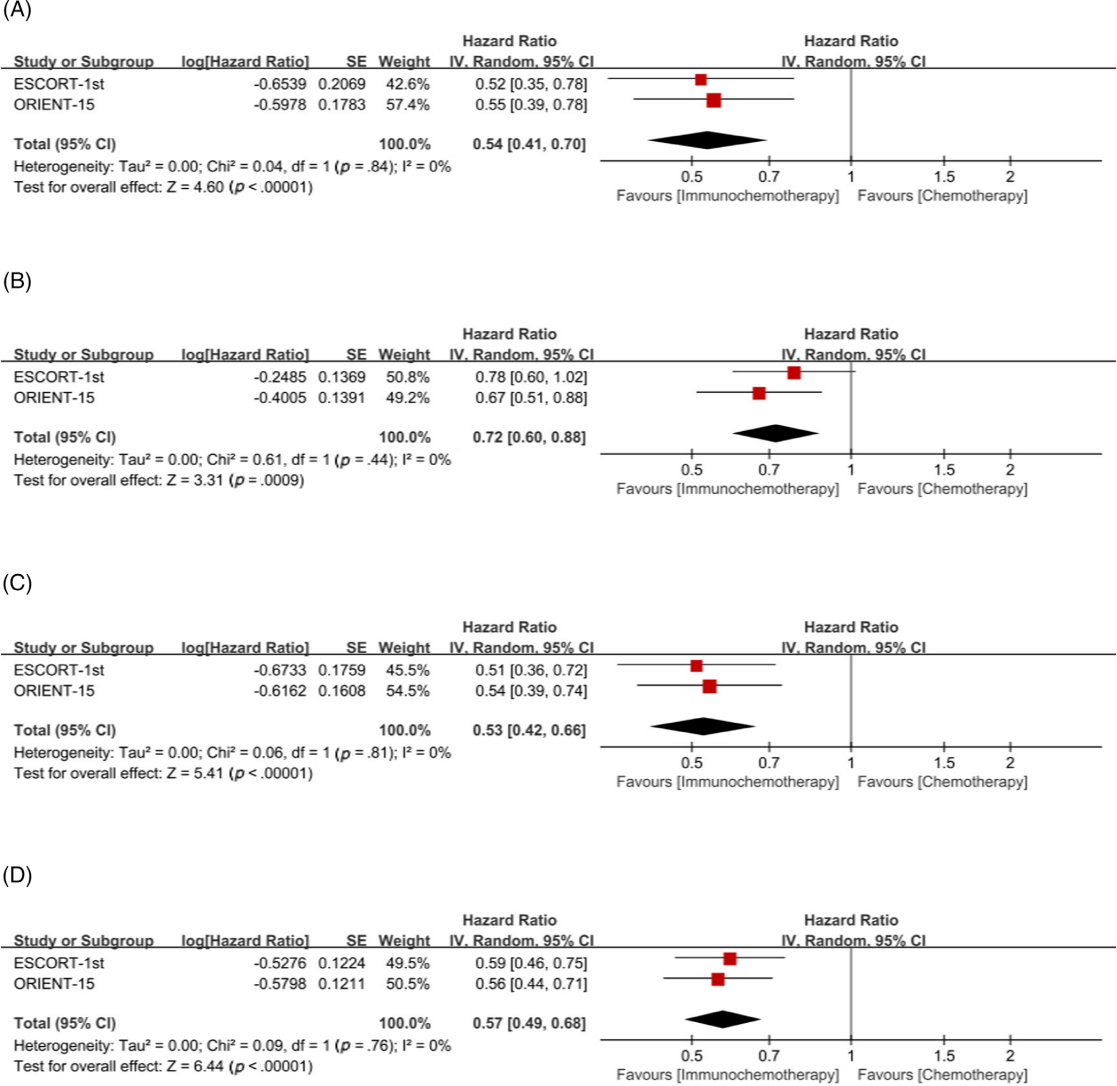
Tumor proportion score (TPS) = 10% was used as cutoff value to classify patients and compare the efficacy of immunochemotherapy and chemotherapy. (A) overall survival (OS) of TPS≥10%; (B) OS of TPS <10%; (C) progression‐free survival (PFS) of TPS≥10%; (D) PFS of TPS <10%.

Subgroup analysis with TPS = 10% as cutoff value, whether TPS≥10% or TPS < 10%, showed that the OS (TPS≥10%: HR = 0.54, 95% CI 0.41–0.70; TPS < 10%: HR = 0.72, 95% CI 0.60–0.88, respectively) (Figure 3A,B) and PFS (TPS≥10%: HR = 0.53, 95% CI 0.42–0.66; TPS < 10%: HR = 0.57, 95% CI 0.49–0.68, respectively) (Figure 3C,D) of patients in the immunochemotherapy group were better than those in the chemotherapy group.

**FIGURE 4:**
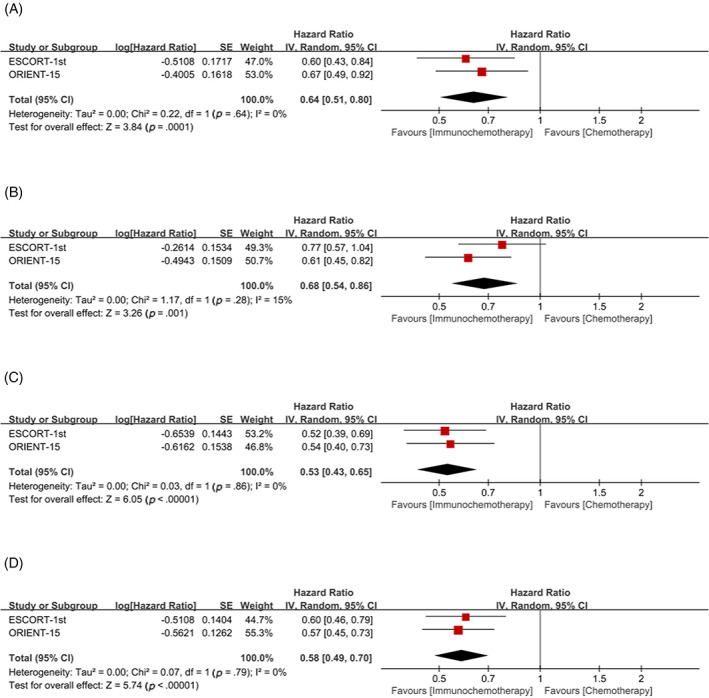
Tumor proportion score (TPS) = 5% was used as cutoff value to classify patients and compare the efficacy of immunochemotherapy and chemotherapy. (A) Overall survival (OS) of TPS≥5%; (B) OS of TPS <5%; (C) progression‐free survival (PFS) of TPS≥5%; (D) PFS of TPS <5%.

Subgroup analysis with TPS = 5% as cutoff value, whether TPS≥5% or TPS <5%, showed that the OS (TPS≥5%: HR = 0.64, 95% CI 0.51–0.80; TPS <5%: HR = 0.68, 95% CI 0.54–0.86, respectively) (Figure 4A,B) and PFS (TPS≥5%: HR = 0.53, 95% CI 0.43–0.65; TPS <5%: HR = 0.58, 95% CI 0.49–0.70, respectively) (Figure 4C,D) of patients in the immunochemotherapy group were better than those in the chemotherapy group.

**FIGURE 5:**
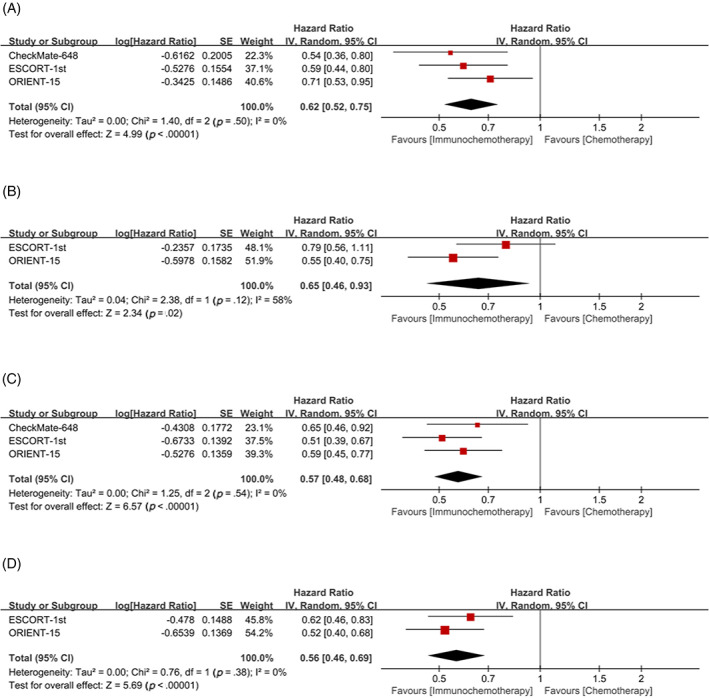
Tumor proportion score (TPS) = 1% was used as cutoff value to classify patients and compare the efficacy of immunochemotherapy and chemotherapy. (A) overall survival (OS) of TPS≥1%; (B) OS of TPS <1%; (C) progression‐free survival (PFS) of TPS≥1%; (D) PFS of TPS <1%.

Subgroup analysis with TPS = 1% as cutoff value, whether TPS≥1% or TPS <1%, showed that the OS (TPS≥1%: HR = 0.62, 95% CI 0.52–0.75; TPS <1%: HR = 0.65, 95% CI 0.46–0.93, respectively) (Figure 5A,B) and PFS (TPS≥1%: HR = 0.57, 95% CI 0.48–0.68; TPS <1%: HR = 0.56, 95% CI 0.46–0.69, respectively) (Figure 5C,D) of patients in the immunochemotherapy group were better than those in the chemotherapy group.

### 3.5 Stratified according to CPS

**FIGURE 6:**
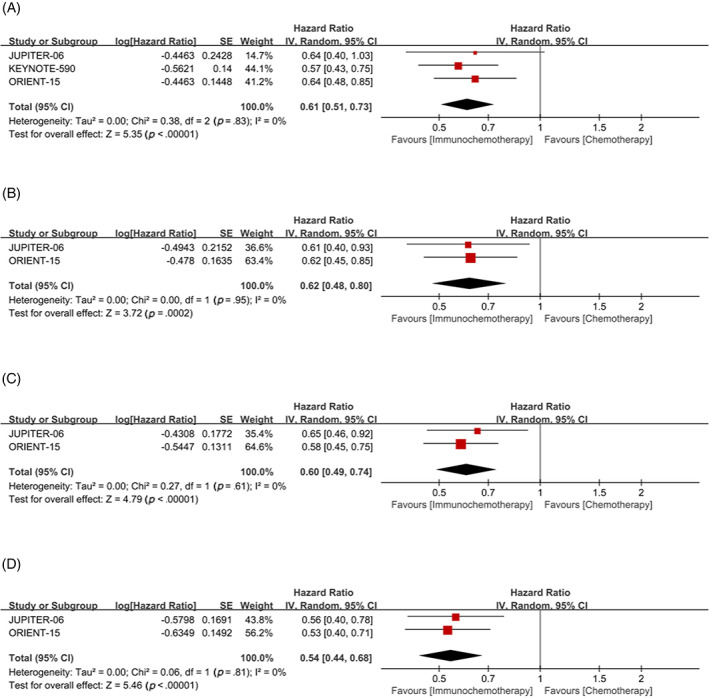
Combined positive score (CPS) = 10 was used as cutoff value to classify patients and compare the efficacy of immunochemotherapy and chemotherapy. (A) Overall survival (OS) of CPS≥10; (B) OS of CPS <10; (C) progression‐free survival (PFS) of CPS≥10; (D) PFS of CPS <10.

Subgroup analysis with CPS = 10 as cutoff value, whether CPS≥10 or CPS <10, showed that the OS (CPS≥10: HR = 0.61, 95% CI 0.51–0.73; CPS <10: HR = 0.62, 95% CI 0.48–0.80, respectively) (Figure 6A,B) and PFS (CPS≥10: HR = 0.60, 95% CI 0.49–0.74; CPS < 10: HR = 0.54, 95% CI 0.44–0.68, respectively) (Figure 6C,D) of patients in the immunochemotherapy group were better than those in the chemotherapy group.

**FIGURE 7:**
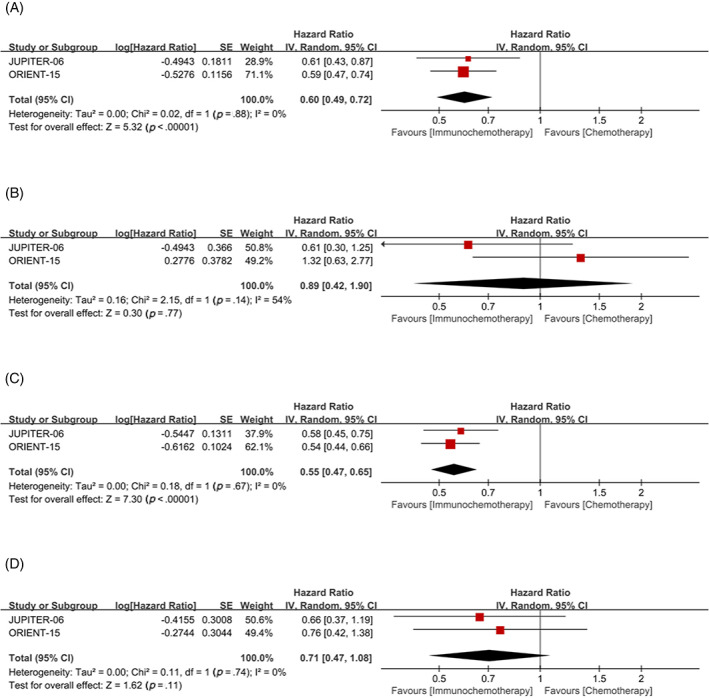
Combined positive score (CPS) = 1 was used as cutoff value to classify patients and compare the efficacy of immunochemotherapy and chemotherapy. (A) Overall survival (OS) of CPS≥1; (B) overall survival (OS) of CPS <1; (C) progression‐free survival (PFS) of CPS≥1; (D) PFS of CPS <1.

Subgroup analysis with CPS = 1 as cutoff value, for patients with CPS≥1, the OS (HR = 0.60, 95% CI 0.49, 0.72) and PFS (HR = 0.55, 95% CI 0.47–0.65) in the immunochemotherapy group were significantly better than those in the chemotherapy group (Figure 7A,C). However, for patients with CPS < 1, the significant benefit of OS (HR = 0.89, 95% CI 0.42–1.90) and PFS (HR = 0.71, 95% CI 0.47–1.08) in the immunochemotherapy group did not exist (Figure 7B,D).

### 3.6 Safety

All clinical trials showed the incidence of the TRAEs and TRM. In addition, KEYNOTE‐590 trial presented the incidence of the TRAEs and TRM for EAC and ESCC, which was included in the meta‐analysis considering that adverse events were mostly multiorgan systemic. Compared with the chemotherapy group, the incidence of any grade or ≥ grade 3 TRAEs in the immunochemotherapy group was higher (OR = 1.74, 95% CI 1.32–2.29; OR = 1.32, 95% CI 1.00–1.75, respectively) (Figure S2A,B). In addition, the incidence of serious TRAEs, any grade irAEs, and ≥ grade 3 irAEs was reported in four studies respectively, and the incidence of immunochemotherapy group was significantly higher than that of chemotherapy group (OR = 1.65, 1.29–2.09; OR = 3.39, 95% CI 1.56–7.36; OR = 3.16, 95% CI 2.05–4.89, respectively) (Figure S2C–E). Although patients in the immunochemotherapy group were more likely to develop TRAEs, there was no significant difference in TRM between the two groups (OR = 1.11, 95% CI 0.67–1.83) (Figure S2F).

### 3.7 Sensitivity analysis

Significant inter‐study heterogeneity was found in the comparison of grade ≥3 TRAEs and any grade irAEs (I^2^ = 70%, *p* = .01; I^2^ = 94%, *p* < .01, respectively) (Figure S2B,D). Sensitivity analyses showed that the greatest heterogeneity came from ESCORT‐1st trial (Figure S3A,B). After omitting the ESCORT‐1st test, OR = 1.47 (95% CI 1.18–1.83) for grade ≥3 TRAEs and OR = 2.30 (95% CI 1.65–3.21) for any grade irAEs (Figure S3A,B).

Moderate heterogeneity was also found in subgroup analyses of OS with TPS <1% and CPS <1 (I^2^ = 58, *p* = .12; I^2^ = 54, *p* = .14, respectively) (Figure 5B, Figure 7B). As only two studies were included, the source of heterogeneity could not be judged. Since the heterogeneity is moderate and the random effect model has been used to avoid the influence of heterogeneity as much as possible, the results have certain reliability.

### 3.8 Publish bias assessment

The egger's funnel plot shows no significant publication bias in OS meta‐analysis, *p* = .50 (Figure S4).

## 4 DISCUSSION

Our study demonstrated that compared with the chemotherapy alone, PD‐1/PD‐L1‐based immunochemotherapy significantly improved survival outcomes in patients with advanced ESCC. More importantly, immunochemotherapy had excellent long‐term efficacy regardless of TPS value. However, in patients with CPS <1, there was no significant survival advantage in patients receiving immunochemotherapy. This could help to provide useful guidance for future research and treatment programs. In addition, TAREs were significantly increased in the immunochemotherapy group compared with the chemotherapy group, but TRM was not significantly different between the two groups.

The treatment of early stage ESCC depends on surgical resection or endoscopic treatment.^28^, ^29^ When the disease progresses to a locally advanced stage, the efficacy of surgery alone is very limited. After the publication of the results of the CROSS and NEOCRTEC5010 trials, preoperative neoadjuvant chemoradiotherapy combined with surgery became the standard treatment for locally advanced resectable ESCC, and patients' postoperative survival was significantly improved.^30^, ^31^ However, for patients with advanced esophageal cancer, surgery has been unable to achieve a radical cure. In terms of survival, surgery has lost its meaning. In addition, traditional treatment methods such as chemotherapy and radiotherapy have limited effect on patients with advanced esophageal cancer, so the treatment of these patients has always been a difficult problem.

In recent 10 years, immune checkpoint inhibitors have become a new entry point for cancer treatment.^6^, ^7^ Currently, three commonly used immune checkpoint inhibitors are anti‐PD‐1, anti‐PD‐L1, and anti‐CTLA4 derivatives. Inhibition of activated CTLA‐4, PD‐1, and PD‐L1 pathways can reverse T helper cell‐mediated immunosuppression. As an epoch‐making groundbreaking treatment, immunotherapy has demonstrated efficacy in many advanced cancers.^6^, ^7^ Immunotherapy based on PD‐1/PD‐L1 has long been applied in the field of esophageal cancer. Many phase I/II trials have proved that immunotherapy is effective as a second‐line or multi‐line treatment for advanced esophageal cancer.^9^, ^10^, ^11^, ^12^, ^13^, ^14^, ^15^ This increased great confidence for immune checkpoint inhibitors to advance into the field of first‐line treatment of advanced esophageal cancer.

Several clinical trials have revealed the results of immune checkpoint inhibitors as a first‐line treatment for advanced esophageal cancer. KEYNOTE‐590 trial (pembrolizumab and chemotherapy (cisplatin/5‐fluorouracil) vs. placebo and chemotherapy) is the first clinical trial to report results of immunochemotherapy as first‐line treatment for locally advanced/unresectable or metastatic adenocarcinoma, ESCC, or Siewert Type 1 gastroesophageal junction adenocarcinoma.^21^ The study showed that the OS of pembrolizumab plus chemotherapy group was significantly longer than that of placebo plus chemotherapy group (12.4 months vs. 9.8 months, HR = 0.73, *p* < .0001), and the benefit of OS was superior in patients with ESCC and CPS≥10 (median survival time: 13.9 vs. 8.8 months; HR = 0.57; *p* < .0001). This study paved the way for pembrolizumab to enter the first‐line treatment of advanced esophageal cancer. On March 22, 2021, the USFDA approved pembrolizumab in combination with platinum and fluorouracil for the first‐line treatment of advanced esophageal cancer and gastroesophageal junction cancers. Since then, the results of various clinical trials have been published. The results of ESCORT‐1st (Camrelizumab), CheckMate‐648 (Nivolumab), ORIENT‐15 (sintilimab), and JUPITER‐06 (toripalimab) trials all showed that for advanced ESCC, the long‐term efficacy of immunochemotherapy as a first‐line treatment regimen was significant than chemotherapy alone.^22^, ^23^, ^24^, ^25^ A meta‐analysis by Leone et al. showed that PD‐1/PD‐L1based immune checkpoint inhibitors significantly reduced CPS ≥10 ESCC patients compared with chemotherapy.^32^ However, the study did not distinguish between immune checkpoint inhibitor monotherapy and a combination of immune checkpoint inhibitor and chemotherapy. Our meta‐analysis showed that immunochemotherapy reduced the overall risk of death by 32% (HR = 0.68, 95% CI 0.61–0.75) and the risk of disease progression by 38% (HR = 0.62, 95% CI 0.55, 0.70) in patients with advanced ESCC compared with chemotherapy alone. In addition, we also conducted an in‐depth study according to the expression level of PD‐L1 in ESCC. When the expression level of PD‐L1 was evaluated by TPS value, we found that the immune checkpoint inhibitors had excellent long‐term efficacy regardless of TPS value, and the long‐term efficacy of immunochemotherapy was significantly better than that of chemotherapy alone. When the expression level of PD‐L1 was evaluated by CPS value, it was found that in patients with CPS <1, although immunochemotherapy reduced the risk of long‐term death and disease progression compared with chemotherapy alone, there was no significant difference (OS: HR = 0.89, 95% CI 0.42–1.90; PFS: HR = 0.71, 95% CI 0.47–1.08, respectively). These results remind us that PD‐1/PD‐L1 pathway inhibitors may not be suitable for all patients, and the cutoff value of PD‐L1 expression level and other characteristic biomarkers still need to be further studied.

To date, no clinical trial was conducted for EAC alone, so we did not analyze this group of patients. Before the KEYNOTE‐590 trial, the CheckMate‐649 trial conducted a comparative study of nivolumab combined chemotherapy and chemotherapy alone in the treatment of gastric cancer, gastroesophageal junction cancer and EAC.^21^, ^33^ Encouragingly, both KEYNOTE‐590 and CheckMate‐649 trials showed that immunochemotherapy improved overall survival of EAC (HR = 0.74, 95% CI 0.54–1.02; HR = 0.82, 95% CI 0.60–1.13, respectively).

While immunotherapy brings hope for survival, TRAEs, especially irAEs, should not be ignored. These have been reported in all cancers, and in severe cases leads to drug withdrawal and even death.^34^, ^35^, ^36^, ^37^ In the clinical trials we included, almost all of the studies reported higher toxicity of immunochemotherapy than chemotherapy alone. The meta‐analysis after the summary confirmed this point: compared with chemotherapy alone, the incidence of any grade TRAEs, grade ≥3 TRAEs, serious TRAEs, any grade irAEs, grade ≥3 irAEs in patients with immunochemotherapy were significantly higher. However, there was no significant difference in TRM between the two treatments. This is not difficult to understand, as the use of immune checkpoint inhibitors becomes more widespread and physicians become better equipped to manage their related adverse reactions.

Although the included studies were prospective RCT_S_ and all were of high quality as assessed by the Cochrane Handbook, limitations remained in this meta‐analysis. First, we did not have access to data on every patient, so we could not do a more detailed analysis. Second, although all the drugs we studied were derived from PD‐1/PD‐L1, the use of chemotherapy drugs was not completely consistent. Third, not all trials reported the study endpoints we wanted to analyze, and some endpoints only included two studies, which would have an impact on the results of the meta‐analysis. Finally, heterogeneity was found in subgroup analysis of OS with TPS <1% and CPS <1 (I^2^ = 58%, *p* = .12; I^2^ = 54%, *p* = .14, respectively) (Figure 5B, Figure 7B). Although we used the random effect model to avoid the effect of heterogeneity as much as possible, the source of heterogeneity could not be analyzed because only two studies were included, so the interpretation of the results should be cautious. Because the heterogeneity of these two subgroup analyses was moderate, the results were somewhat convincing.

## 5 CONCLUSIONS

The treatment‐related mortality of immunochemotherapy was similar to that of chemotherapy. Overall, PD‐1/PD‐L1 immunochemotherapy significantly improved long‐term survival outcomes in patients with advanced ESCC compared with chemotherapy, independent of PD‐L1 TPS status but associated with CPS status. When CPS <1, there was no significant difference between the efficacy of immunochemotherapy and chemotherapy. Further investigations of biomarkers for immunochemotherapy in the subgroup of patients with CPS <1 are needed.

## AUTHOR CONTRIBUTIONS

**Zixian Jin:** Data curation (equal); formal analysis (equal); investigation (equal); methodology (equal); software (equal); validation (equal); visualization (equal); writing – original draft (equal); writing – review and editing (equal). **Jiping Wang:** Data curation (equal); formal analysis (equal); investigation (equal); methodology (equal); software (equal); validation (equal); visualization (equal); writing – original draft (equal). **Jiajing Sun:** Data curation (equal); formal analysis (equal); investigation (equal); methodology (equal); software (equal); validation (equal); visualization (equal); writing – original draft (equal). **Chengchu Zhu:** Writing – review and editing (equal). **Jian Zhang:** Conceptualization (equal); methodology (equal); project administration (equal); resources (equal); supervision (equal); validation (equal); writing – review and editing (equal). **Bo Zhang:** Conceptualization (equal); methodology (equal); project administration (equal); resources (equal); supervision (equal); validation (equal); writing – review and editing (equal).

## FUNDING INFORMATION

None.

## CONFLICT OF INTEREST STATEMENT

The authors declare that they have no competing interests.

## ETHICS STATEMENT

Not applicable.

## Supporting information


**Supplementary Table S1.** Risk of Bias Assessment: Based on the Cochrane Handbook.
**Supplementary Figure 1.** Flow chart of literature retrieval and screening.
**Supplementary Figure 2.** Forest plot of safety comparison between immunochemotherapy and chemotherapy. (A) Any grade TRAEs; (B) ≥3 grade TRAEs; (C) serious TRAEs; (D) Any grade irAEs; (E) ≥3 grade irAEs; (F) TRM.
**Supplementary Figure 3.** Sensitivity analysis of ≥3 grade TRAEs and any grade irAEs. (A) ≥3 grade TRAEs; (B) Any grade of irAEs.
**Supplementary Figure 4.** The egger's funnel plot for overall survival.

## Data Availability

Data sharing is not applicable.
