# Supplementary material for: PD‐1/PD‐L1 based immunochemotherapy versus chemotherapy alone for advanced esophageal squamous cell carcinoma: A meta‐analysis focus on PD‐L1 expression level
Source: Cancer Rep (Hoboken). 2023 May 18;6(7):e1794. doi: 10.1002/cnr2.1794 (PMC10363809; doi:10.1002/cnr2.1794)
Supplement: Supplementary file 1 — Supplementary Table S1. Risk of Bias Assessment: Based on the Cochrane Handbook. Supplementary Figure 1. Flow chart of literature retrieval and screening. Supplementary Figure 2. Forest plot of safety comparison between immunochemotherapy and chemotherapy. (A) Any grade TRAEs; (B) ≥3 grade TRAEs; (C) serious TRAEs; (D) Any grade irAEs; (E) ≥3 grade irAEs; (F) TRM. Supplementary Figure 3. Sensitivity analysis of ≥3 grade TRAEs and any grade irAEs. (A) ≥3 grade TRAEs; (B) Any grade of irAEs. Supplementary Figure 4. The egger's funnel plot for overall survival. [file CNR2-6-e1794-s001.docx]

**Supplementary Table 1.** Risk of Bias Assessment: Based on the Cochrane Handbook.

| Study | random sequence generation | allocation hiding | blindness of participants and personnel | blindness of result evaluations | incomplete result data | selective result reporting* | other sources of bias |
| --- | --- | --- | --- | --- | --- | --- | --- |
| KEYNOTE-590 | + | + | + | + | + | ？ | + |
| CheckMate-649 | + | + | ？ | + | + | ？ | + |
| ESCORT-1st | + | + | + | + | + | ？ | + |
| CheckMate-648 | + | + | ？ | + | + | ？ | + |
| ORIENT-15 | + | + | + | + | + | + | + |
| JUPITER-06 | + | + | + | + | + | ？ | + |

*Reporting bias due to selective reporting of results. The ORIENT-15 trial reported all our endpoints and was assessed as low risk according to the Cochrane Handbook.

**Supplementary Figure 1.** Flow chart of literature retrieval and screening.


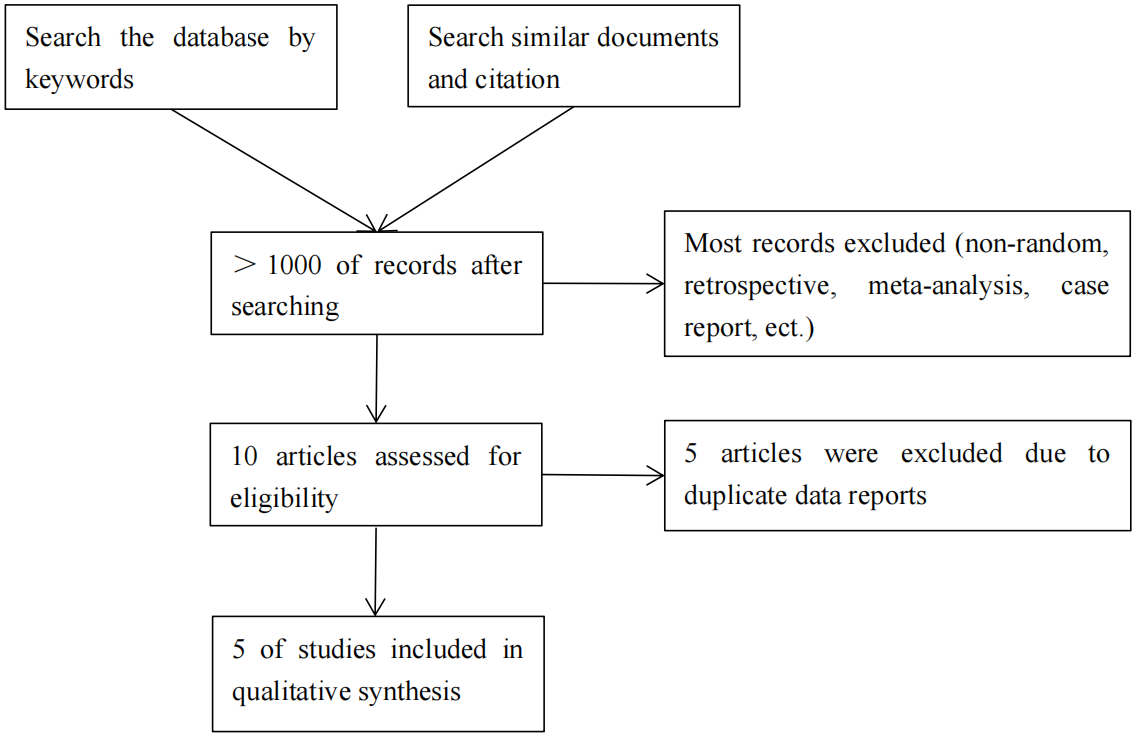


**Supplementary Figure 2.** Forest plot of safety comparison between immunochemotherapy and chemotherapy. (A) Any grade TRAEs; (B) ≥3 grade TRAEs; (C) serious TRAEs; (D) Any grade irAEs; (E) ≥3 grade irAEs; (F) TRM.

(A)


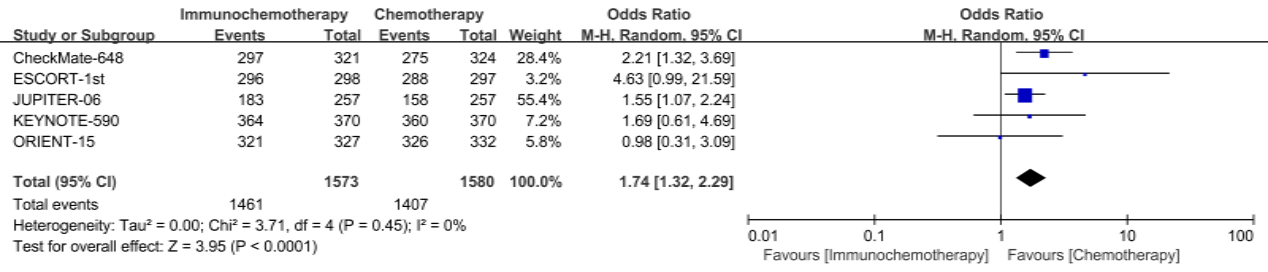


(B)


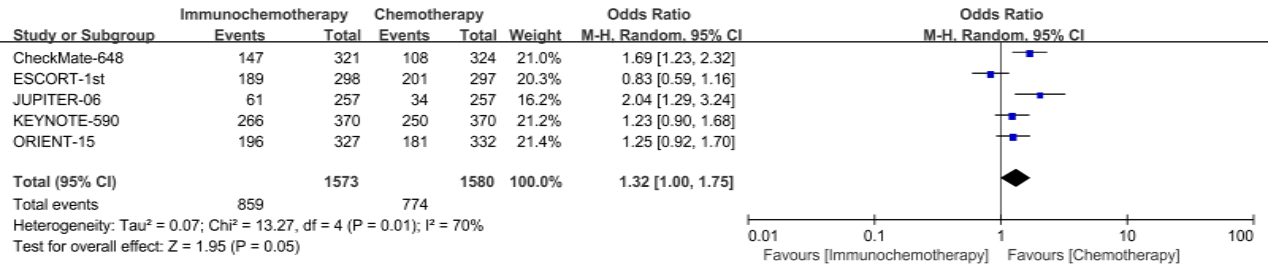


(C)


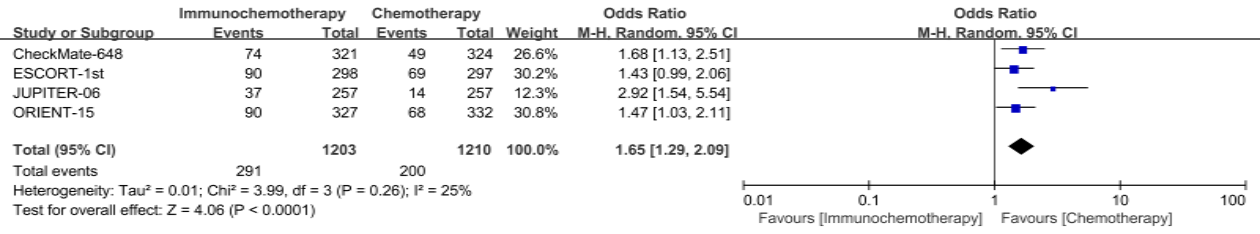


(D)


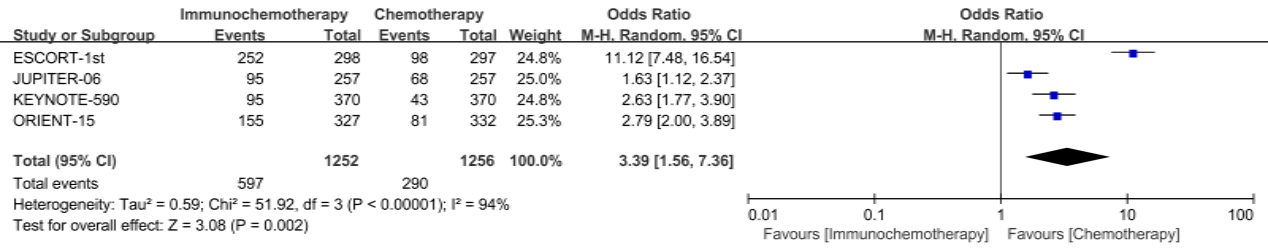


(E)


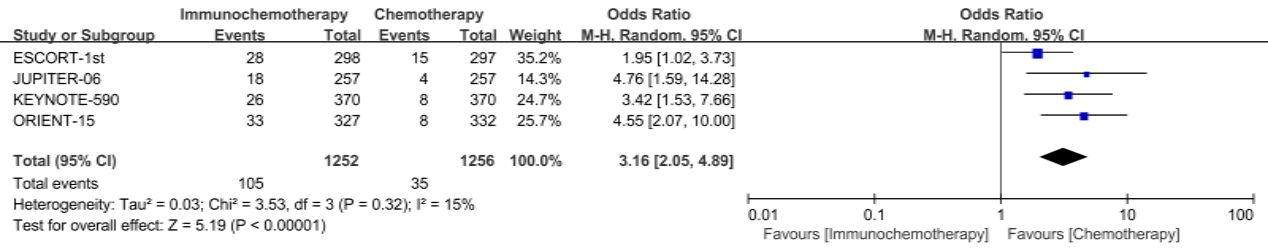


(F)


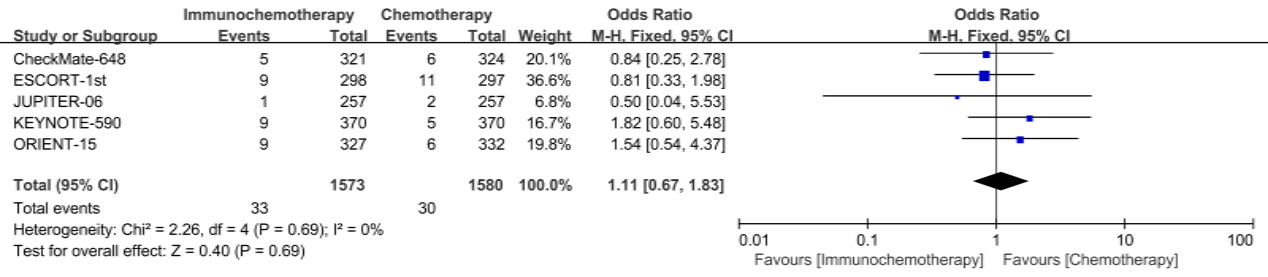


**Supplementary Figure 3.** Sensitivity analysis of ≥3 grade TRAEs and any grade irAEs. (A) ≥3 grade TRAEs; (B) Any grade of irAEs.

(A)


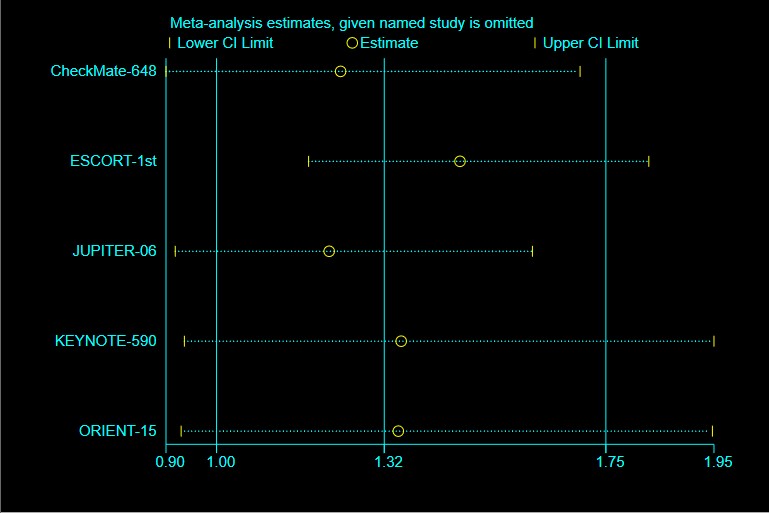

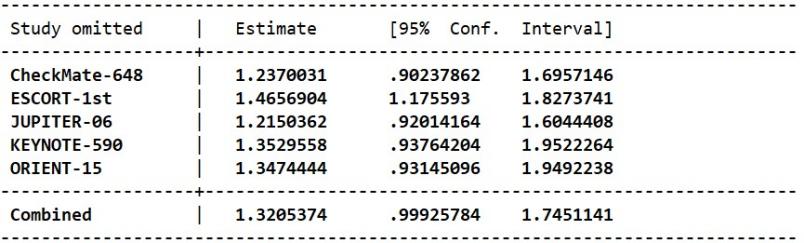


(B)


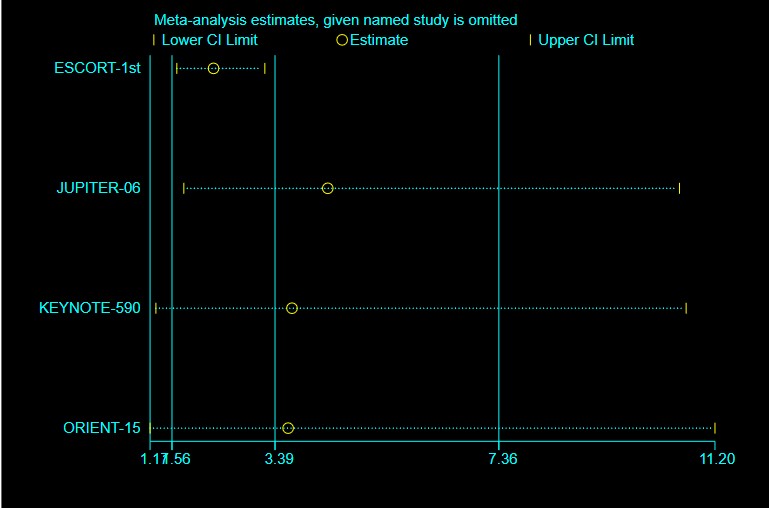

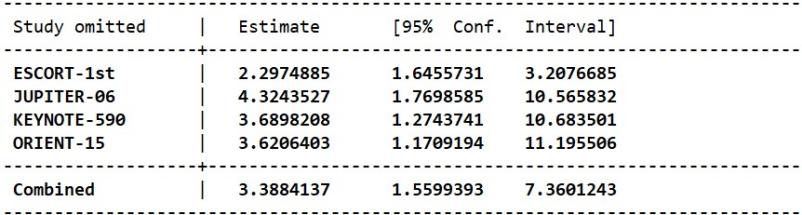


**Supplementary Figure 4.** The egger's funnel plot for overall survival.


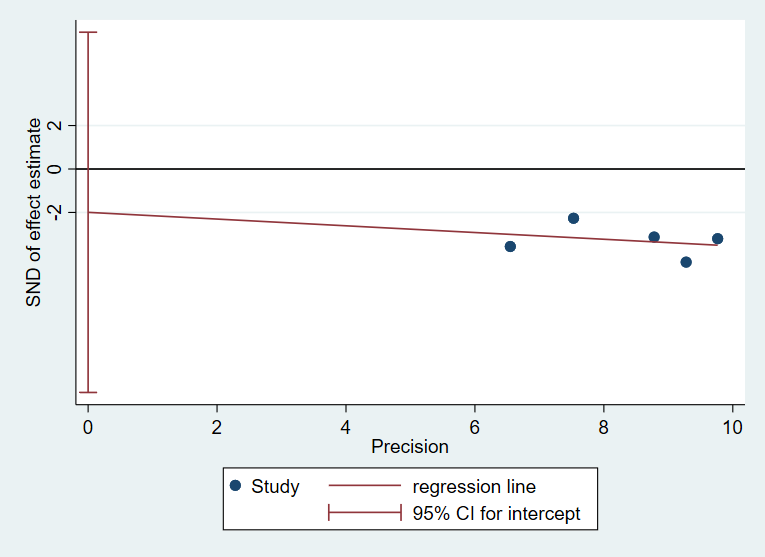


P=0.50
